# Mechanical, Fatigue, and Thermal Characterization of ASA, Nylon 12, PC, and PC-ABS Manufactured by Fused Filament Fabrication (FFF)

**DOI:** 10.3390/polym18020302

**Published:** 2026-01-22

**Authors:** Ângela Rodrigues, Ricardo Branco, Margarida Franco, Rui Silva, Cândida Malça, Rui F. Martins

**Affiliations:** 1 UNIDEMI, Department of Mechanical and Industrial Engineering, NOVA School of Science and Technology, Campus de Caparica, 2829-516 Caparica, Portugal; amc.rodrigues@campus.fct.unl.pt; 2 CEMMPRE, ARISE, Department of Mechanical Engineering, University of Coimbra, Rua Luís Reis Santos, Pinhal de Marrocos, 3030-788 Coimbra, Portugal; ricardo.branco@dem.uc.pt; 3 Centre for Rapid and Sustainable Product Development, Polytechnic Institute of Leiria, Rua de Portugal, 2430-028 Marinha Grande, Portugal; margarida.franco@ipleiria.pt (M.F.); rui.d.silva@ipleiria.pt (R.S.); candida@isec.pt (C.M.); 4 Department of Mechanical Engineering, Coimbra Polytechnic—ISEC, Rua Pedro Nunes, 3030-199 Coimbra, Portugal; 5 Laboratório Associado de Sistemas Inteligentes, LASI, 4800-058 Guimarães, Portugal

**Keywords:** additive manufacturing, ASA, PC, Nylon 12, PC-ABS, fatigue, micro-CT, TGA, DSC, FTIR

## Abstract

Additive manufacturing has been widely adopted in industry as an alternative to traditional manufacturing processes for complex component production. In fact, a diverse range of materials, particularly polymers, can be processed using 3D printing for biomechanical applications (e.g., prosthetics). However, in-depth evaluation of these materials is necessary to determine their suitability for demanding applications, such as those involving cyclic loading. Following previous work that studied Polylactic Acid (PLA) and Polyethylene Terephthalate Glycol-modified (PETG) under experimental fatigue testing, this study examines the fatigue behaviour of other current 3D-printed polymeric materials, namely Acrylonitrile Styrene Acrylate (ASA), Polycarbonate (PC), Polyamide 12 (Nylon 12), and Polycarbonate–Acrylonitrile Butadiene Styrene (blend) (PC-ABS), for which fatigue data remain limited or even non-existent. The findings revealed performance differences on Tensile Strength (σ_R_), Young’s Modulus and Ultimate Strain among tensile specimens made from these materials and characterised S-N curves for both high-cycle (HCF) and low-cycle (LCF) fatigue regimes at room temperature, with a tensile load ratio (R = 0.05). These results establish relationships among fatigue limit and *quasi*-static mechanical properties, namely 25% × σ_r_ for ASA (8 MPa), 7% × σ_r_ for PC (3.6 MPa), 17% × σ_r_ for Nylon 12 (7.4 MPa), and 15% × σ_r_ for PC-ABS (4.7 MPa), as well as between mechanical properties and preliminary potential biomechanical applications. Main conclusions were further supported by micro-computed tomography (micro-CT), which revealed levels of porosity in between 4% and 11%, thermogravimetric analysis (TGA), differential scanning calorimetry (DSC), and Fourier transform infrared spectroscopy (FTIR).

## 1. Introduction

Following previous work that studied PLA and PETG under experimental fatigue testing [1], this study examines the fatigue behaviour of other current 3D-printed polymeric materials, namely ASA, PC, Nylon 12, and PC-ABS, for which fatigue data remain limited or even non-existent, as summarised in Table 1.

Additive manufacturing is a technology used to produce components by building three-dimensional parts layer by layer, eliminating the need for moulds, turning or milling. In contrast to traditional subtractive manufacturing processes, additive manufacturing enables the rapid production of complex geometries with minimal material waste [2], which contributes to its cost-effectiveness. This technology is widely adopted in industry, particularly in design for manufacturing and assembly applications. Additionally, additive manufacturing can produce a diverse range of parts from various materials, including thermoplastics, metals, ceramics, and composites [3]. Material extrusion, also referred to as Fused Filament Fabrication (FFF) [4], is among the most widely used processes. Therefore, this study focuses primarily on the FFF process.

A primary advantage of the FFF process is, as mentioned above, the extensive range of materials available for part fabrication, with new materials introduced regularly, supporting broad applicability across multiple industrial sectors. However, the process also presents drawbacks, including relatively low surface finish quality, which is critical for fatigue resistance. Because material is deposited layer by layer, small strings and material residues may form during printing, particularly when the nozzle moves between distant points on the part. During these movements, the filament may continue to extrude in small amounts, resulting in undesired strings. Additionally, voids may develop due to the arrangement of deposited layers, reducing component density, and manufacturing speed is strongly influenced by printer parameters, such as layer height and infill density, which can increase production time. Furthermore, the mechanical properties of printed materials vary with the filament deposition direction (anisotropy), and warping is a common issue. In fact, residual stresses form due to temperature differences experienced by the material during deposition and non-uniform cooling, leading to shrinkage and warpage. This often manifests as separation of the part edges from the build platform, resulting in deformations [5,6]. To address this, most printers are equipped with a build platform with adjustable temperature, which is crucial for controlling part cooling and heating [7]. The primary function of this parameter is to ensure effective adhesion between the first deposited filaments. It is essential that the glass transition temperature of the material is similar than the build platform temperature; otherwise, warping may occur, compromising the part’s stability and integrity [8,9,10].

Additionally, polymeric materials used in additive manufacturing are classified by chemical structure as either amorphous or semicrystalline. Amorphous materials, such as ASA, ABS (Acrylonitrile Butadiene Styrene), PETG, PC, and PC-ABS, are characterised by a disordered arrangement of polymer chains, resulting in lower mechanical strength. In contrast, semicrystalline materials, including PLA, Nylon, and PEEK (Polyether Ether Ketone), possess a more organised polymer chain structure with chains aligned along a primary direction, which enhances mechanical strength [11]. Although a wide range of materials can be processed using material extrusion, thermoplastics are most commonly used because they become viscous and malleable at specific temperatures [12]. Table 1 presents experimental results from a literature review of tensile and fatigue tests conducted on each material under study.

The four polymers under investigation were chosen because they are high-performance thermoplastics that are relatively easy to print, cost-effective, suitable for engineering applications, including biomechanical ones, and for which limited information exist under cyclic loading (fatigue).

In fact, the current literature on additively manufactured ASA, PC, PC-ABS, and Nylon 12 is notably limited regarding their fatigue resistance (Table 1), particularly under comparable testing conditions, and most available studies are focused on static mechanical properties, such as tensile or flexural strength, while fatigue behaviour, which is critical for components subjected to cyclic loading in real applications, has received considerably less attention. Furthermore, the existing fatigue data (Table 1) lack a systematic and comparative assessment conducted under a consistent experimental protocol. This does not allow designers and engineers to carry out an informed material selection for applications where fatigue is critical. Hence, the present investigation addresses this gap by providing a consistent, comparative, and detailed dataset on the fatigue behaviour of ASA, PC, PC-ABS, and Nylon 12 under controlled and comparable conditions, thereby extending the existing literature and contributing to a deeper understanding of the fatigue behaviour of newly additively manufactured engineering thermoplastics.

This specific set of polymers, namely ASA, PC, PC-ABS, and Nylon 12, was selected because it represents a group of widely used engineering thermoplastics with overlapping application domains but distinctly different polymer chemistries. Together, they cover amorphous, semi-crystalline, and polymer-blend systems, enabling a meaningful comparative assessment of fatigue behaviour that cannot be achieved by studying materials with similar molecular structures. Moreover, these polymers are extensively used in industrial and additive manufacturing applications where cyclic loading is critical. In addition, PEEK, PETG, and PLA have been previously studied. Hence, the aim was to provide fatigue behaviour data for representative materials with dissimilar molecular structures to support informed material selection by designers and engineers.

ASA is an amorphous thermoplastic material used in a wide range of applications, such as exterior components in the automotive sector. It has emerged as an alternative to ABS and exhibits superior resistance to UV radiation, maintaining its integrity and avoiding colour changes even when exposed to UV radiation for long periods.

PC is an amorphous thermoplastic polymer known for its good impact resistance and high transparency. It is frequently used to manufacture components that require better mechanical properties than materials such as ASA or ABS, particularly in terms of impact resistance (fracture toughness).

Nylon 12 is a semi-crystalline, hygroscopic thermoplastic material widely used in engineering applications. It is characterised by good impact resistance and high toughness. In addition, it is known for its flexibility and ductility, allowing bending without easy fracture and exhibiting high elongation. Nylon 12 also shows good chemical resistance and good electrical insulation properties.

PC-ABS is a polycarbonate-ABS blend that combines the properties of PC and ABS. It is an amorphous thermoplastic cost-effective material that provides a good surface finish and is a toughness-optimised blend.

**Table 1:** *Quasi*-static and fatigue properties of materials under study: Tensile Strength [MPa], σ_R_, Yield Strength [MPa], σ_y_, Young’s Modulus [MPa], E, and Fatigue limit [MPa], σ_f0_. Stress range, Δσ [MPa] and Number of cycles, N.

Material	Type of Test	Printing Parameters	Results [MPa]	No. of Specimens
ASA [13,14]	Uniaxial Tensile tests	Nozzle diameter: 0.4 mm Extrusion temperature: 240 °C Build platform temperature: 60 °C Raster angle: 45° Infill density: 100% Infill pattern: Rectilinear Printing speed: 30 mm/s	$\sigma_{R}=16.82\pm 0.31$	5
PC [15]	Uniaxial Tensile tests	Nozzle diameter: 0.4 mm Extrusion temperature: 275 °C Build platform temperature: 110 °C Infill density: 100% Layer thickness: 0.2 mm	$\sigma_{R}=61.6\;E=305.9$	3
PC [16]	Uniaxial Tensile tests	Nozzle diameter: 0.4 mm Extrusion temperature: 275 °C Build platform temperature: 115 °C Raster angle: multiple values Infill density: 100% Infill pattern: Rectangular Printing speed: 30 mm/s	$\sigma_{R}=48.8\;to\;55.2$	13
		Layer thickness: multiple values		
Nylon 12 [17]	Uniaxial Tensile tests	Extrusion temperature: 270 °C Build platform temperature: 90 °C Raster angle: 45°	$\sigma_{R}=32.2\pm 2.7$	-
		Infill density: 100% Printing speed: 40 mm/s	E=109.5±9.0	
		Layer thickness: 0.2 mm		
Nylon 12 [18]	Uniaxial Tensile tests	Extrusion temperature: 255 °C Build platform temperature: 85 °C Raster angle: 45°	$\sigma_{R}=32.9\pm 2.5$	5
		Infill density: 100% Printing speed: 40 mm/s	E=153.5±9.6	
		Layer thickness: 0.2 mm		
Nylon 12 [19]	Fatigue	SLS manufactured	Basquin Law: Load applied parallel to deposition layers: $\Delta \sigma =111.1\times N^{-0.11}$	69
			$R^{2}=0.76$	
			Load applied perpendicular to deposition layers:	
			$\Delta \sigma =83.1\times N^{-0.09}$	
			$R^{2}=0.55$	
PC-ABS [20]	Uniaxial Tensile tests	-	$\sigma_{R}=52.7\pm 0.6$	6
	Fatigue	-	$\sigma_{f0}=45.2\pm 0.6$	

As stated previously, Table 1 indicates that research on fatigue behaviour in fused filament fabrication (FFF)-printed polymeric materials remains limited. Furthermore, fatigue failure predominantly occurs in filaments near areas with non-uniform layer bonding, such as voids, and under continuous cyclic loading, these conditions facilitate plastic deformation and promote crack propagation along the polymers’ filaments [21]. Additionally, uniaxial tensile tests have shown a decreasing trend in tensile strength for PC/PC-ABS, Nylon, and ASA materials, in that order.

Polymer components produced by additive manufacturing through material extrusion have become increasingly significant in biomechanics. This approach has gained traction in the biomechanical services by enabling the development of cost-effective, efficient prototypes. However, not all polymers are suitable for use within the human body. In fact, a material is defined as biocompatible only when it demonstrates compatibility with the host organism. Surface compatibility involves the effective integration of the implant with body tissues, considering chemical, physical, and biological factors, whereas structural compatibility pertains to how the implant material fits and performs mechanically with both soft and hard tissues. Therefore, prosthetic materials must exhibit mechanical properties, such as elastic modulus, ultimate tensile strength, and yield strength, which are compatible with the surrounding tissues [22,23].

For example, during routine activities, bones typically experience stresses of approximately 4 MPa, whereas tendons and ligaments are subjected to stresses of 40 to 80 MPa. The hip joint, for example, can withstand average loads of about 3000 N, with this value increasing substantially during high-impact activities such as jumping, where forces may reach up to ten times the body weight [22]. Additionally, S. Ramakrishna et al. estimate that finger and hip joints undergo more than 1 × 10^6^ cycles annually [22].

Georg Bergmann et al. [24] also analysed the forces and moments acting on hip implants, emphasizing the importance of ISO standards and recommending that fatigue tests be performed at frequencies between 1 Hz and 30 Hz. For external-use prostheses, Merel van der Stelt et al. [25] investigated the additive manufacturing of tibial prostheses using Tough PLA. Fatigue tests at 2 Hz demonstrated that these prostheses could endure 2.27 × 10^6^ cycles under a load of 1.2 kN. Tensile tests further indicated a maximum supported load of 6700 N [25].

Studies examining forces and induced stresses in dentistry also report that the compressive stress range in mandibular trabecular bone is 0.22 to 10.44 MPa [26]. The force applied during mastication typically ranges from 50 to 260 N, with a frequency range of 1.2 to 6 Hz. However, estimates of the number of mastication cycles per year vary widely, ranging from 2.5 × 10^5^ to 1 × 10^6^ cycles [27].

Therefore, fatigue behaviour is a critical consideration for both internal and external prostheses, as well as biomedical devices produced by additive manufacturing for human use.

## 2. Materials and Methods

### 2.1. Materials

Table 2 presents the technical specifications of the materials tested, including their commercial name, grade, color, glass transition temperature, and density. All feedstock materials, namely ASA, PC-ABS, Nylon 12, and PC, were supplied in filament form by Stratasys Ltd. (Eden Prairie, MN, USA).

### 2.2. Specimens

The production of the ASA, PC, Nylon 12, and PC-ABS specimens was carried out using a Stratasys Fortus 450 printer (Stratasys Ltd., Eden Prairie, MN, USA). Table 3 presents the printing parameters used for the specimens.

**Figure 1:**
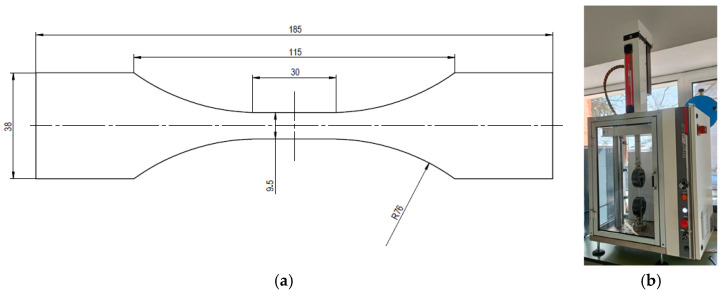
(**a**) The 3D-printed specimens (dimensions are in millimetres); (**b**) STEP Lab EA05 testing machine.

Specimens for tensile and fatigue testing were prepared in accordance with ASTM D638-03 [28] and Figure 1a presents the specimen dimensions. All specimens had a thickness of approximately 5.2 mm.

### 2.3. Uniaxial Quasi-Static Monotonic Tensile Tests and Fatigue Tests

Uniaxial *quasi*-static monotonic tensile tests and fatigue tests were performed using a STEP Lab EA05 electromechanical machine (Figure 1b). The *quasi*-static monotonic tensile tests employed a displacement rate of 2 mm/min, while the fatigue tests were conducted at 10 Hz with a load ratio (R) of 0.05 and a load-controlled sinusoidal waveform. The load cell capacity was 10 kN for *quasi*-static tests and 5 kN for fatigue tests.

The staircase (up-and-down) method was used to determine the fatigue limit values for all tested materials. Initially, higher stress levels were applied to obtain finite fatigue lives. Subsequently, the stress levels were decreased to identify fatigue life near 1,000,000 cycles (run-out). Stress levels were then increased again to assess the new finite fatigue life. This process was repeated until the fatigue limit and remaining fatigue resistance values were established for all the tested materials.

### 2.4. Micro-CT

Computed tomography (CT) is a non-destructive technique that uses X-rays to produce images of a sample’s internal structure. From these images, it is possible to study, for example, voids and porosity, and, in the case of FFF samples, to determine the printing orientation used as well [29]. Micro-computed tomography (micro-CT) analyses were conducted using a Skyscan 1174 system (Bruker Corporation, Billerica, MA, EUA).

### 2.5. TGA and DSC

Thermogravimetric analysis (TGA) is a widely used thermal analysis technique for assessing the thermal stability and properties of polymers. This type of analysis records the mass variation in a sample when subjected to high temperatures, often associated with the corresponding material degradation. On the other hand, differential scanning calorimetry (DSC) is mainly used to evaluate phase transitions, such as melting or glass transition [30,31]. Thermal tests were conducted using an STA 6000 instrument (PerkinElmer, Inc. Shelton, CT, EUA). Samples were heated from 30 °C to 800 °C at a rate of 10 °C per minute. The tests were performed under an inert nitrogen atmosphere with a flow rate of 20 mL per minute. An aluminium oxide sample holder was employed during the analyses.

### 2.6. FTIR

Fourier transform infrared spectroscopy (FTIR) is an analytical technique frequently used to identify the presence and distribution of various functional and structural groups. This technique is based on the absorption of infrared radiation by molecules at specific frequencies, corresponding to the vibrational modes of their chemical bonds [32,33]. The FTIR analyses were carried out using an FTIR-ATR Alpha P instrument (Bruker Corporation, Billerica, MA, EUA).

### 2.7. Scanning Electron Microscopy (SEM)

The fracture surfaces of the specimens tested under fatigue loading for the four additively manufacture materials were examined using scanning electron microscopy to analyse the fracture topography and identify the main failure characteristics associated with the different 3D-printed polymers. The analysis was carried out using a Carl-Zeiss Gemini 500 field emission scanning electron microscope (Carl Zeiss AG, Oberkochen, Germany). Before examination by SEM, the samples were ultrasonically cleaned for 10 min and then sputter-coated with a thin layer of gold to prevent charging during imaging.

## 3. Results and Discussion

### 3.1. Uniaxial Quasi-Static Monotonic Tensile Tests

**Figure 2:**
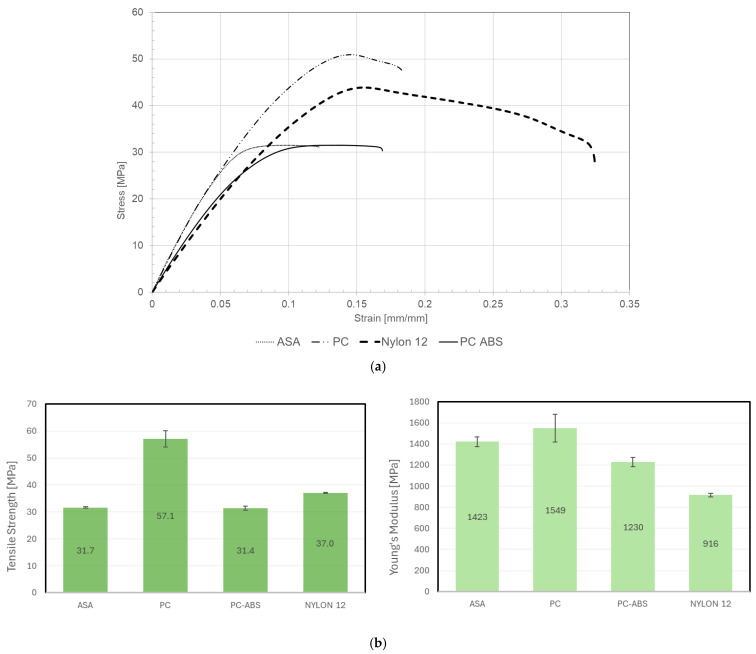
(**a**) Typical stress–strain curves obtained in the uniaxial *quasi*-static tensile tests carried out for the four materials under study, namely ASA, PC, Nylon 12 and PC-ABS; (**b**) Mechanical properties of the tested materials.

Uniaxial tensile tests were conducted to determine the *quasi*-static properties of the materials. The initial cross-sectional area of the ASA, PC, Nylon 12, and PC-ABS specimens was about 49 mm^2^, and the initial length considered for l_0_ was 30 mm. Figure 2 shows an example of stress–strain curves obtained for each material tested, and Table 4 presents the values of Young’s modulus, and tensile strength obtained from the uniaxial tensile tests carried out on the four materials. A total of five specimens were tested for each material under study.

Analysis of Figure 2 and the data presented in Table 4 indicate that all tested materials exhibit ductility, with values exceeding around 6%. Nylon 12 demonstrates the highest ductility, surpassing 30%. Moreover, based on the areas under the stress–strain curves, in the elastic and plastic regimes, it can be concluded that all materials display resilience and toughness, respectively, with Nylon identified as the toughest polymer, followed by polycarbonate (PC). The ASA and PC-ABS polymers exhibit mechanical behaviour characteristic of perfectly elastic-plastic materials. Polycarbonate (PC) is the strongest polymer, followed by Nylon, ASA, and PC-ABS.

### 3.2. Fatigue Tests

As previously mentioned, all fatigue tests were carried out at 10 Hz with a load ratio (R) of 0.05, using the staircase (up-and-down) method to determine the fatigue limit values for all tested materials. Moreover, each specimen’s cross-section was measured before testing to determine the maximum and minimum forces to be applied. The tests always started with high-stress-range (Δσ) values, defined relative to the Tensile Strength (σ_R_) of each material (Table 3), in the low-cycle fatigue (LCF) regime (<10^4^ cycles). Thereafter, the applied stress range values were progressively reduced to determine the Basquin Law in the high-cycle fatigue (HCF) regime (>10^4^ cycles) and the fatigue limit for each material tested (1 × 10^6^ cycles, run-out). The definition of run-out for 1,000,000 cycles is also consistent with the biomechanical loading cycles cited in Section 1.

#### 3.2.1. ASA

**Figure 3:**
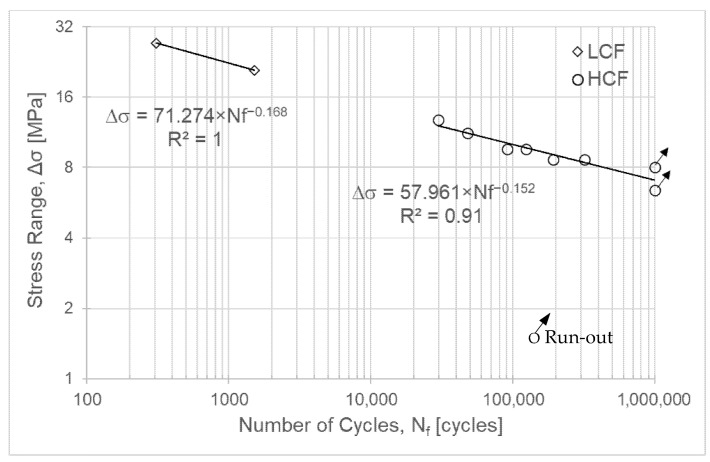
S-N curve for ASA.

The testing parameters for ASA are presented in Table 5, and the S-N fatigue curves are shown in Figure 3. For the first specimen, a stress range (Δσ) corresponding to 85% of the ultimate tensile strength (σ_R_) of the ASA material was defined. The number of cycles obtained was low (308), so the applied stress range was subsequently reduced.

Specimens 4 and 5 (Table 5) exhibited a fatigue life of 1,000,000 cycles and can thus be considered to have infinite life (run-out).

Figure 3 presents the S-N curve for the ASA material. The values on the *Y*-axis are displayed on a base-2 logarithmic scale to facilitate reading. Conversely, a base-10 logarithmic scale was chosen for the *X*-axis values, as is typically used for S-N curves. Thus, the behaviour of ASA at low cycle fatigue and high cycle fatigue can be described by Equations (1) and (2), respectively.

(1) $${\Delta \sigma }_{LCF}=71.274{{\;\times \;N}_{f}}^{-0.168}$$

(2) $${\Delta \sigma }_{HCF}=57.961{\;\times \;N_{f}}^{-0.152}$$

#### 3.2.2. PC

**Figure 4:**
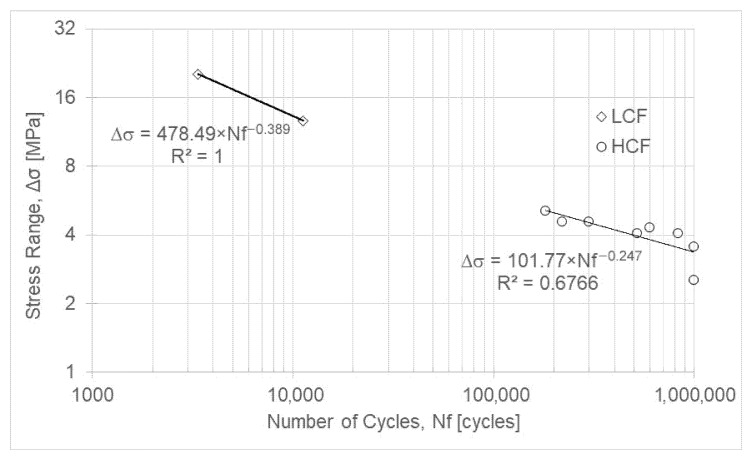
S-N curve for PC.

The selection of the percentages used to define the stress range was based on the same criteria applied to the ASA material. For the first specimen tested, a 40% tensile-strength criterion was chosen (Table 6), allowing assessment of the number of cycles to failure. Based on this result, the strategy for the subsequent tests was to significantly reduce the stress range percentage to determine the threshold at which the material fails by fatigue or exhibits infinite life. PC exhibited the lowest fatigue strength among the tested materials (Table 6, Figure 4). In addition, a significant variation is noted between the 8% and 9% of the tensile strength. Therefore, two specimens were tested for each of these percentages, and one specimen was tested at the intermediate value of 8.5%.

Equations (3) and (4) represent the fatigue life of PC for low-cycle fatigue and high-cycle fatigue, respectively.

(3) $${\Delta \sigma }_{LCF}=478.49{{\;\times \;N}_{f}}^{-0.389}$$

(4) $${\Delta \sigma }_{HCF}=101.77{\;\times \;N_{f}}^{-0.247}$$

#### 3.2.3. Nylon-12

**Figure 5:**
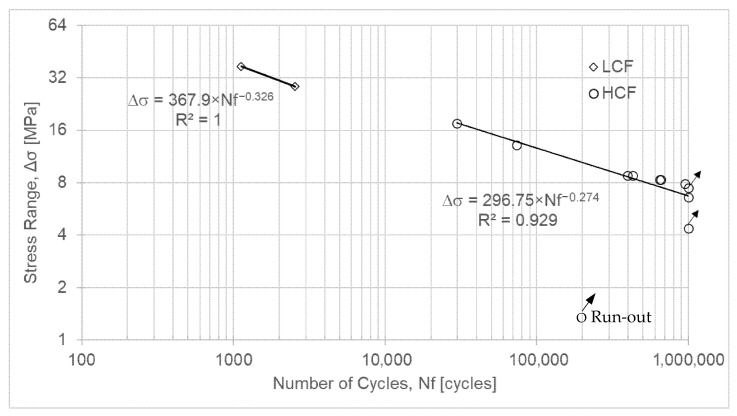
S-N curve for Nylon 12.

It should be noted that, as observed for PC, this material exhibits a very narrow range of variation (Table 7, Figure 5). Infinite life lies between the threshold values of 17% and 18% of σ_R_. It is observed that the three specimens tested at load levels below 17% of σ_R_ did not fail by fatigue. However, it is worth noting that there is a marked difference in the number of cycles obtained within the 18% to 20% range.

Equations (5) and (6) represent the fatigue life of Nylon-12 for low-cycle fatigue and high-cycle fatigue, respectively.

(5) $${\Delta \sigma }_{LCF}=367.9{{\;\times \;N}_{f}}^{-0.326}$$

(6) $${\Delta \sigma }_{HCF}=296.75{\;\times \;N_{f}}^{-0.274}$$

#### 3.2.4. PC-ABS

**Figure 6:**
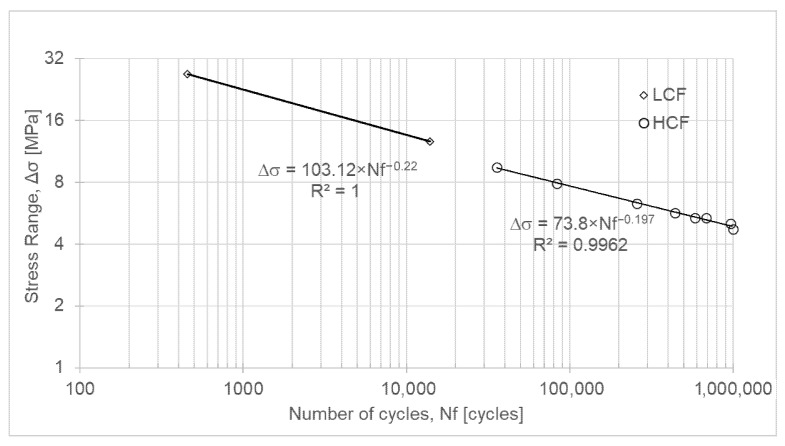
S-N curve for PC-ABS.

It should be noted that this material, composed of PC and ABS, exhibits fatigue resistance properties (Table 8, Figure 6) superior to those observed for the PC material (Table 6, Figure 4). Thus, with the addition of ABS, the material has a longer fatigue life.

Equations (7) and (8) represent the fatigue life of PC-ABS for low-cycle fatigue and high-cycle fatigue, respectively.

(7) $${\Delta \sigma }_{LCF}=103.12{{\;\times \;N}_{f}}^{-0.22}$$

(8) $${\Delta \sigma }_{HCF}=73.8{\;\times \;N_{f}}^{-0.197}$$

### 3.3. Micro-CT

**Figure 7:**
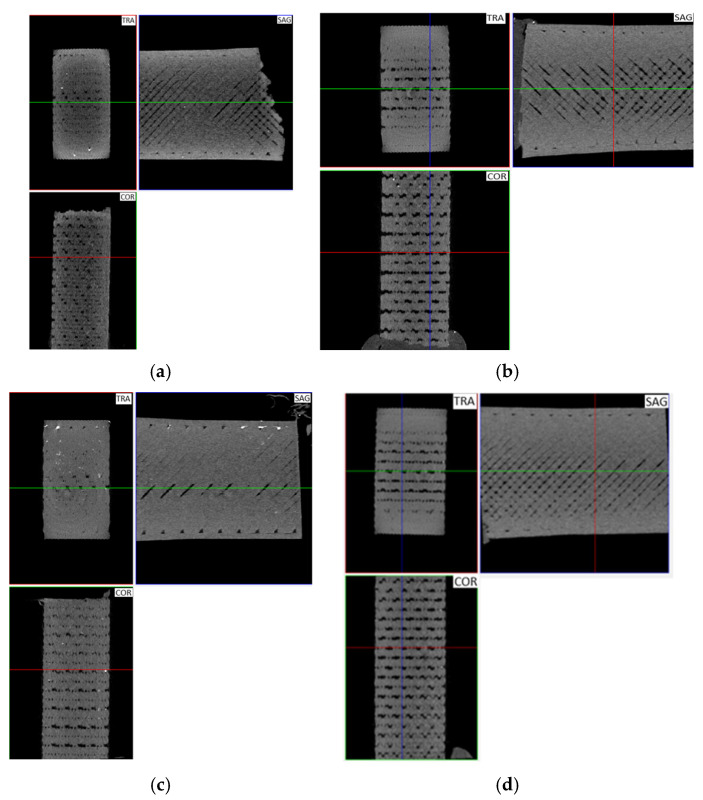
Micro-CT analyses: (**a**) ASA; (**b**) PC; (**c**) Nylon 12; (**d**) PC-ABS.

From the micro-CT analysis of the polymers under study, it is possible to identify in the SAG views (Figure 7) that the infill angle is 45°/−45° and that, although the specimens were produced with a 100% infill density, regions with some voids between the filaments can be observed. In the TRA and SAG views, a higher concentration of voids is observed at the centre of the sample. This may have promoted crack propagation in this region during fatigue and indicates that alternative printing orientations should be adopted to obtain structures at the centre of components that are as dense as those at their periphery and contour.

Furthermore, the printing orientation of the specimens is clearly identifiable as the XY plane; that is, the specimens were printed horizontally, with growth along the vertical *Z*-axis.

Table 9 shows the amount of porosity observed in each sample. Note that this table presents the porosity values obtained for each polymer under study. In addition, to compare the porosity percentages obtained for each polymer, the same sample volume was used for all materials. The porosity ranged from 4.2% for Nylon 12 to 11% for polycarbonate (PC), which helps to explain the differences in fatigue life among the various materials and PC’s relatively low fatigue life. Therefore, it may be helpful to correlate the estimate fatigue limit of additively manufactured materials with their porosity level [34].

### 3.4. TGA and DSC

These thermal analysis techniques, thermogravimetric analysis (TGA) and differential scanning calorimetry (DSC), were carried out for all the materials under study. Table 10 summarizes all the results obtained. Materials such as PC and PC-ABS show a graphical interpretation like that of the ASA material. However, it should be highlighted that Nylon 12, due to being a semi-crystalline polymer, exhibits a different DSC curve.

**Figure 8:**
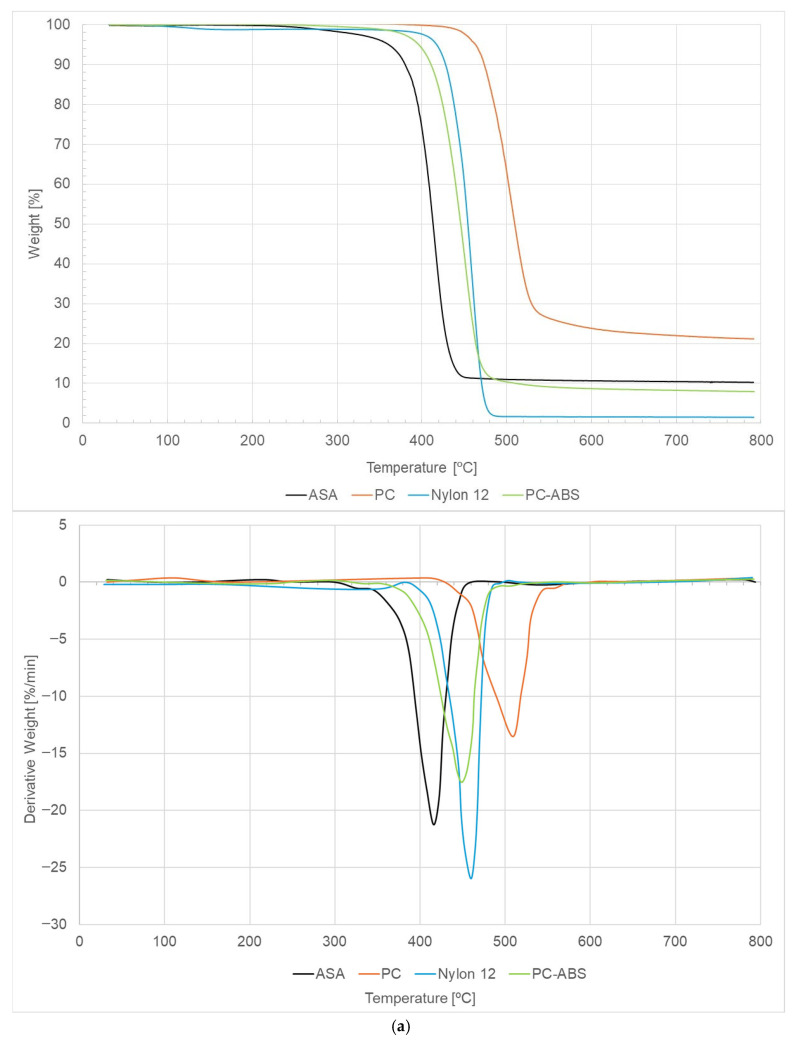

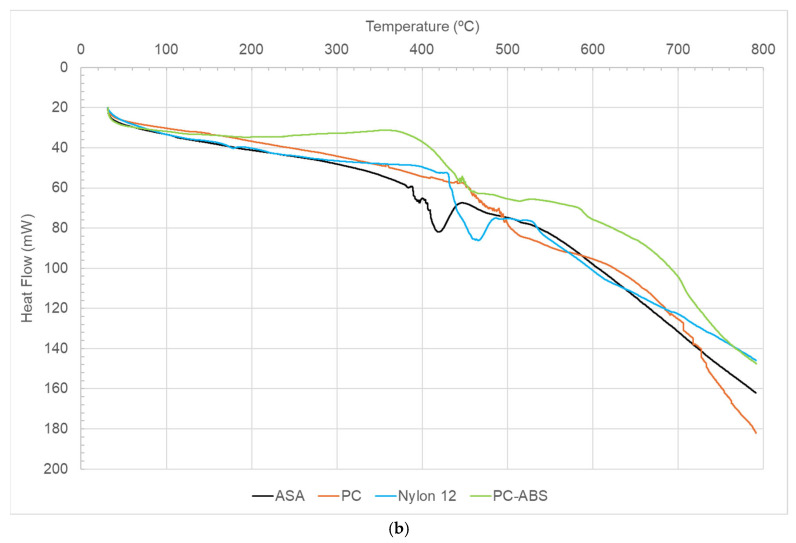
ASA, PC, Nylon 12 and PC-ABS: (**a**) TGA curves; (**b**) DSC curves—Onset temperature of final degradation (ASA: 420.12 °C; PC: 505.95 °C; Nylon 12: 462.21 °C; PC-ABS: 447.71 °C).

Thermogravimetric analysis (TGA) (Figure 8a) and differential scanning calorimetry (DSC) results (Figure 8b) indicate that all studied materials (ASA, PC, PC-ABS, and Nylon 12) exhibit thermal degradation temperatures above 400 °C (Table 10). This confirms that the selected printing and testing conditions do not cause thermal degradation that could affect mechanical or fatigue performance and establishes the limiting design and operating temperatures.

Additionally, PC shows the highest degradation temperature, indicating superior thermal stability and a wider safety margin for high-temperature applications, whereas ASA, Nylon 12 and PC-ABS present slightly lower degradation temperatures but still sufficiently high for engineering use (Table 10).

Concerning the mass loss rate, it reflects the kinetics of thermal degradation. Therefore, materials with higher absolute mass loss rates, such as Nylon 12 and ASA (Table 10), degrade more rapidly once degradation begins, which may imply greater sensitivity to prolonged thermal exposure or thermal ageing. Conversely, lower mass loss rates, such as those occurring on PC, suggest a more gradual degradation process, better supporting high temperature and showing improved thermal durability for a long-term period.

Nevertheless, it is important to note that all materials tested in this investigation are thermally stable within the studied processing window.

### 3.5. FTIR

The result of this analysis is generally presented as a graph: the *X*-axis shows the wavelength (cm^−1^), which indicates the frequency of the infrared light absorbed, while the *Y*-axis shows the absorbance, i.e., the amount of light absorbed at that frequency. The peaks in the graph indicate where molecules absorb energy to vibrate, allowing identification of chemical bonds [32,33]. It is important to emphasise that FTIR analysis is an essential tool, e.g., in biomedicine. This technique enables, for example, the study of tissues to identify molecular changes associated with diseases, such as osteoporosis. In addition, it allows analysis of the chemical bonds of the main molecules that constitute bone, such as phosphate, carbonate, and amides I, II, and III.

**Figure 9:**
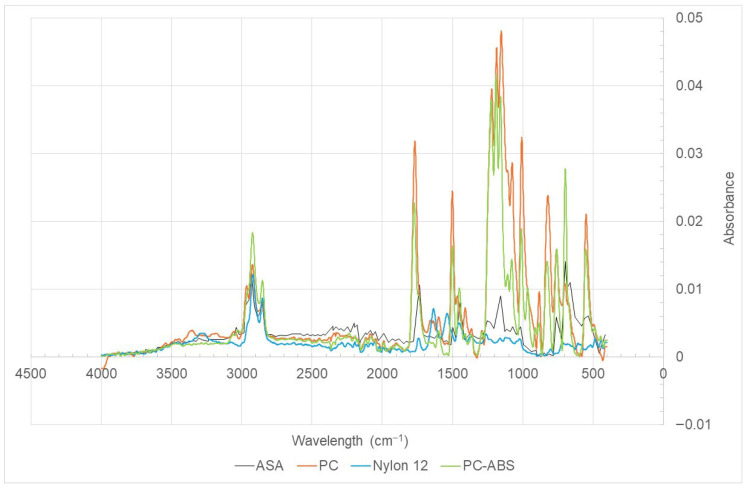
Nylon 12, ASA, PC, PC-ABS. FTIR analyses.

The aim of this analysis was to relate the peaks observed in the FTIR spectrum to characteristic wavelength ranges to determine the functional groups present in each specimen. Figure 9 shows the results obtained for the polymers under study.

As Nylon 12 is a potential biocompatible polymer, a preliminary verification was made between the chemical bonds of bone and those of the polymer. Nikolaos et al. [35] report that the amide I band occurs in the 1600–1720 cm^−1^ range, with C=O as its functional group. Amide II lies in the 1500–1600 cm^−1^ region, corresponding to a combination of the C–N and N–H functional groups. Finally, they also refer to the 1300–1220 cm^−1^ range as being attributed to amide III, which is based on the same functional groups as amide II. Some similarities can be highlighted between the peaks observed in Figure 9 and those of bone structure:

- The amide I band appears at a value close to 1650 cm^−1^. In Figure 9, a peak with a similar value, 1636 cm^−1^, is observed;
- Amide II presents a value close to 1550 cm^−1^. In the FTIR analysis of Nylon 12 (Figure 9), a similar peak at 1540 cm^−1^ was identified;
- Amide III appears at a value of approximately 1240 cm^−1^. A similar peak is also present in Nylon 12 at 1261 cm^−1^.

For the remaining polymers, i.e., ASA, PC, and PC-ABS, this comparison will only be relevant once their cytotoxicity and, therefore, biocompatibility with the human body have been confirmed.

### 3.6. SEM Analyses

**Figure 10:**
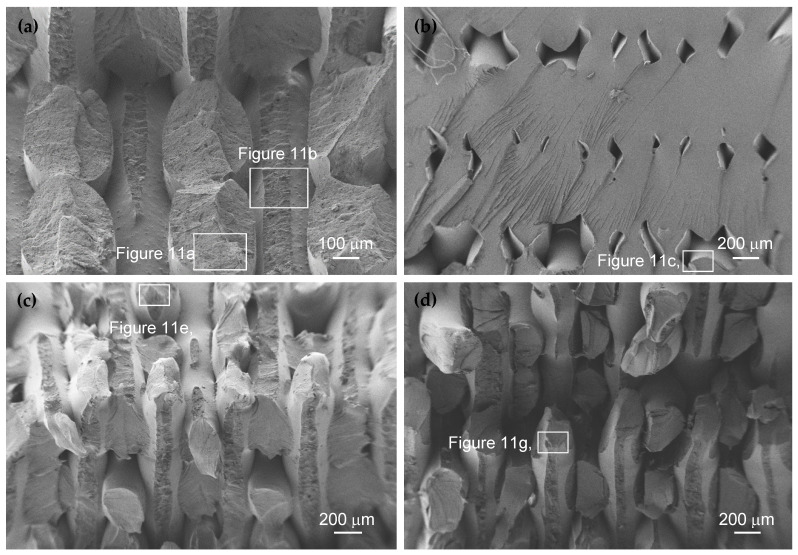
Scanning electron microscopy images of fracture surfaces caused by fatigue loading: (**a**) ASA; (**b**) PC; (**c**) Nylon-12; and (**d**) PC-ABS. The areas outlined in white are magnified in Figure 11.

**Figure 11:**
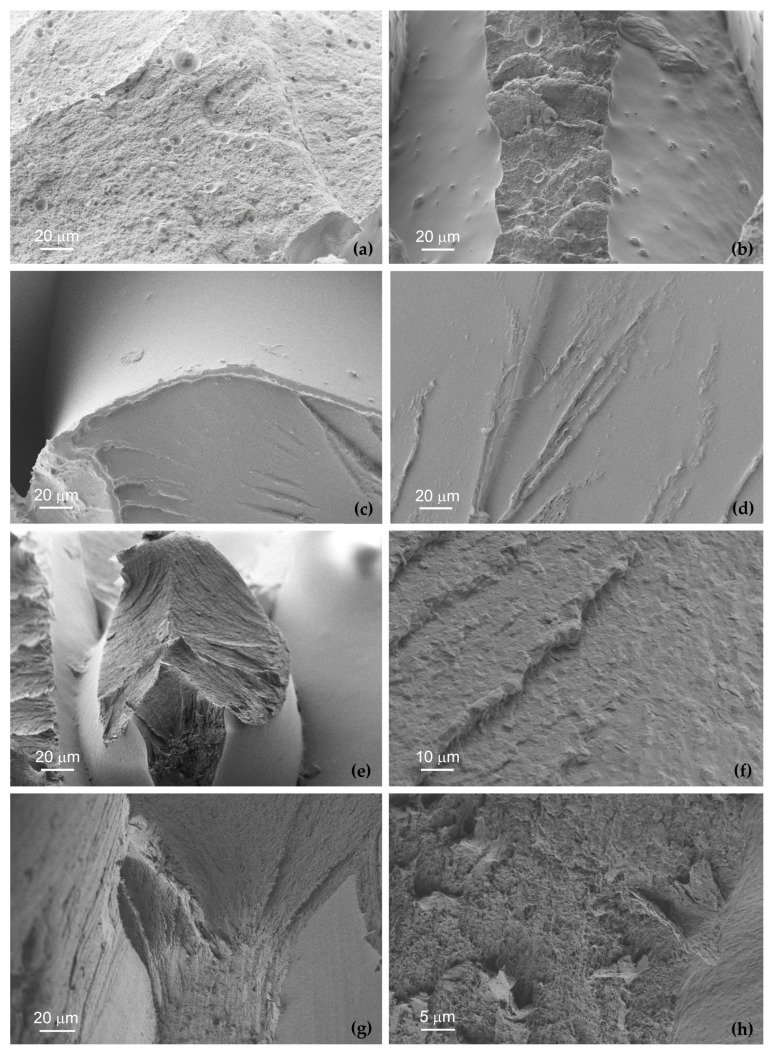
High-magnification SEM images of fracture surfaces caused by fatigue loading: (**a**,**b**) ASA; (**c**,**d**) PC; (**e**,**f**) Nylon-12; and (**g**,**h**) PC-ABS.

Figure 10 and Figure 11 display typical SEM images of fracture surfaces of the tested polymers caused by fatigue loading. Figure 10 shows an overview of the typical appearance of fracture surfaces while Figure 11 shows high-magnification micrographs of the additively manufactured materials investigated in this study.

Regarding the overview images, see Figure 10, it is possible to observe satisfactory interlayer diffusion and good bonding between adjacent extruded filaments along the build layers, indicating adequate thermal fusion during the material deposition process.

In general, the filament pairing pattern associated with the selected raster strategy can be clearly inferred from the micrographs. Overall, some randomly distributed internal voids are observed on the fracture surfaces, particularly in the PC specimens, where the defects appear more systematically and exhibit larger sizes, in agreement with the X-ray computed tomography observations discussed above.

These internal voids are associated with the deposition of adjacent filaments by the imposed hatch distance, highlighting the critical role of the printing strategy and the selected processing parameters (including the hatch spacing, extrusion temperature, and deposition speed) in controlling the internal defect formation. Such voids can also significantly influence the mechanical response of additively manufactured polymers, and particularly the fatigue performance, since they reduce the effective load-bearing cross-section, lowering the cyclic tensile-compressive strength and stiffness. Moreover, they can act as potential fatigue crack initiation sites due to the localised stress–strain concentrations within the material, reducing its suitability for applications subjected to cyclic loading. The presence of voids can also lower fracture toughness, increasing the susceptibility to unstable and unpredictable failure.

Although porosity is detrimental for structural applications requiring high strength and high durability, additively manufactured polymers with controlled amount of voids can be suitable for applications where lightweight design is a priority and mechanical loading is relatively moderate. Previous studies have correlated the fatigue performance with the population of defects, more precisely their size and location, and have shown that optimising processing parameters and improving interlayer bonding can substantially enhance the mechanical properties and fatigue resistance of additively manufactured polymers [1,34,36,37]. Similar conclusions were reported by Martins et al. [1] regarding the improvement of fatigue performance in 3D-printed PLA and PETG materials.

The different fracture surface morphologies of the tested additively manufactured materials can be more clearly observed in the magnified micrographs presented in Figure 11. Regarding the ASA material, the typical fracture surface shown in Figure 10a reveals two distinct failure mechanisms associated with the filament orientation relative to the loading direction. Figure 11a shows filaments failing through their cross-sections, while Figure 11b shows a decohesion mechanism between filaments and regions with incomplete fusion, which is consistent with the typical failure mechanisms reported for this additively manufactured polymer [38]. Furthermore, it is also possible to observe characteristic porosity with variable size and depth dispersed across the entire fracture surface, particularly in Figure 11a.

Concerning the PC material, voids with different sizes and shapes were observed across the entire fracture surface, see Figure 10b. Figure 11c shows, in detail, one of these so-called opened-up crazes, with a characteristic V-shaped morphology, which indicates plastic deformation prior to the final failure. Their presence on the fracture surface, predominantly located along interfilament boundaries, suggests craze initiation followed by the breakdown of fibrillar bridges, promoted by limited interlayer bonding and ultimately leading to crack formation [39]. Figure 11d exhibits a relatively flat fracture region with converging line patterns, often called river patterns, which correspond to crack propagation paths generally caused by craze growth and coalescence. These converging markings indicate the direction towards the crack initiation region and reflect the fracture mechanisms of PC.

As far as the Nylon-12 is concerned, the failure mechanisms are similar to those reported for ASA. Figure 11e displays a typical example of a filament failing through its cross-section, as well as a failure mode caused by decohesion between adjacent filaments. Filaments primarily failed through shear-dominated mechanisms and filament pull-out, with localised plastic deformation contributing to the overall fracture. Figure 11f shows beach marks which are associated with the fatigue crack propagation stage and highlight the ductile fatigue response of the material under cyclic loading.

In relation to the PC-ABS material, it is possible to observe, see Figure 11h, two distinct types of failure within the same filament (see Figure 11h and the white-outlined area of Figure 10d): failure through the filament cross-section; and interlayer failure resulting in decohesion between adjacent filaments. A high magnification of this region, as shown in Figure 11h, reveals a complex fracture surface topography, characterised by irregular dimples with varying diameters and shapes, interconnected by numerous ligaments. These ligaments appear to facilitate the stress distribution and energy absorption, contributing to enhanced ductility compared to pure PC, resulting in improved mechanical performance [40].

## 4. Discussion and Future Work

### 4.1. Experimentally Supported Main Findings

**Figure 12:**
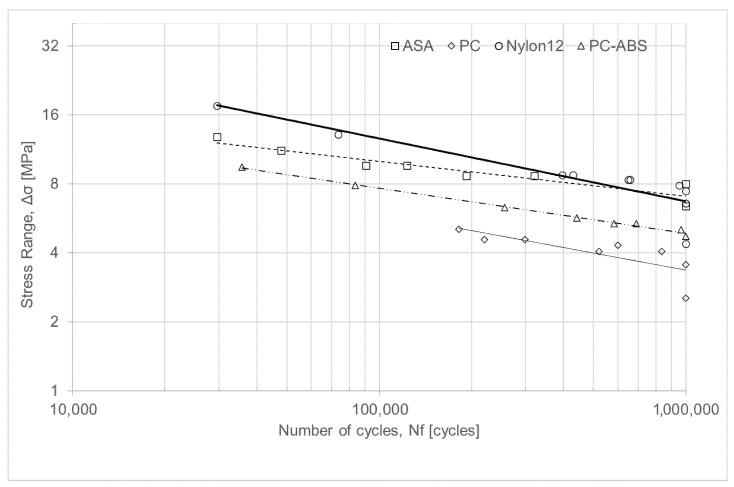
Comparison of mean S-N curves (50% survival probability) for the high-cycle fatigue regime of Nylon 12, ASA, PC, and PC-ABS.

As previously seen (Section 3), it was possible to describe the fatigue behaviour of the materials under study for high-cycle fatigue (HCF) using Basquin’s law and to obtain the corresponding S-N curves (Figure 12). In addition, the experimental programme was extensive, and the resulting fatigue dataset, particularly for ASA and PC, is of interest due to the limited availability of such data in the literature (Table 1). In addition, the study is limited to a single printing orientation (±45°), a single stress ratio (R = 0.05, i.e., representative of pulsating-type loading), and a single loading frequency (10 Hz, which, as discussed in the Introduction section, may arise under biomechanical loading conditions). Nevertheless, for transparency, it is important to reiterate the loading conditions applied in this investigation, which may need adjustment for conditions beyond those studied. Moreover, the ±45° orientation is particularly beneficial for structural components subjected to complex loading, such as tension-bending or bending-torsion, and to cyclic loading (fatigue), where fatigue resistance and damage tolerance are more critical than maximum strength along a single direction, as it activates shear deformation mode, reduces the likelihood of brittle failure along a single interface/filament, and leads to a more quasi-isotropic in-plane behaviour.

Concerning the study of low-cycle fatigue (LCF), it was not the main focus of this investigation, since the objective was to understand the durability of polymers for long-term biomechanical applications. Nevertheless, unlike what frequently occurs in metals, the slopes of the lines relating the stress range to the number of cycles to fracture obtained for ASA, Nylon 12, and PC-ABS (S-N curves), in the LCF and HCF regimes, were practically the same and shared the same intercept, for each of these materials, suggesting the existence of a single fatigue resistance line in the two aforementioned regimes, LCF and HCF. Indeed, only for the PC material did the slopes and intercepts differ between the LCF and HCF regimes. Therefore, for ASA, Nylon 12, and PC-ABS, the fatigue strength decreases continuously with the number of cycles, without an abrupt change in behaviour between LCF and HCF, indicating that the dominant fatigue damage mechanism is essentially the same in both regimes (LCF and HCF). Therefore, unlike what frequently occurs in metals, where LCF is dominated by plastic deformation and HCF by elastic behaviour, ASA, Nylon 12 and PC-ABS do not exhibit a clear transition in damage mechanism between the two regimes.

On the other hand, Table 11 presents the percentages of the ultimate tensile strength, σ_R_, together with the corresponding results and the maximum force values from which the materials exhibited infinite life. ASA, Nylon 12, and PC-ABS can reach infinite life when subjected to stresses below 25% × σ_R_, 17% × σ_R_, and 15% × σ_R_, respectively. However, PC proved to be a material with poor resistance to cyclic loading, reaching infinite life only at stresses below 7% × σ_R_, which is certainly related to the higher porosity measured in specimens manufactured from this material. In addition, from Figure 12, it is possible to conclude that Nylon 12 is the most fatigue-resistant material compared to the other materials investigated in this study, that the fatigue limit value of Nylon 12 and ASA are approximately the same, and that Nylon 12, PC, and PC-ABS revealed almost the same slope in the Basquin Law.

Additionally, by comparing the experimental results obtained for Nylon 12 with those reported in the literature [19], see Table 1, it can be concluded that the specimens tested in the present study yielded higher fatigue strength values than those previously reported. Indeed, the experimentally determined intercept of the Basquin curve in the HCF regime (296.8 MPa), as shown in Figure 5, is higher than the 111.1 MPa reported in [19], while the slope of the line (−0.274) is steeper than the −0.110 reported in [19], see Table 1. Concurrently, about the fatigue limit stress of PC-ABS, the experimentally obtained value (4.7 MPa), see Figure 6, is one order of magnitude lower than that reported in [20], see Table 1.

Moreover, the fatigue strength of components produced by fused filament fabrication (FFF) is determined by microstructural aspects inherent to the production process, including interlayer bonding quality, raster-induced anisotropy, and the tendency for crack initiation and propagation along filament interfaces. In fact, insufficient interlayer bonding, caused by insufficient diffusion between adjacent layers, results in weak interfaces that serve as crack-nucleation sites that can grow under fatigue loading. Furthermore, printing strategies, namely different raster angles, can introduce mechanical anisotropy, leading to stress concentrations when the loading direction is misaligned with the filament orientation. Therefore, beyond nucleation, fatigue crack growth predominantly propagates along inter-bead and interlayer boundaries, where reduced cohesion and increased porosity promote crack propagation and accelerate failure. These failure mechanisms were observed in the experimental fatigue tests (Section 3) and were confirmed through scanning electron microscopy (SEM) analyses (Section 3.6).

**Figure 13:**
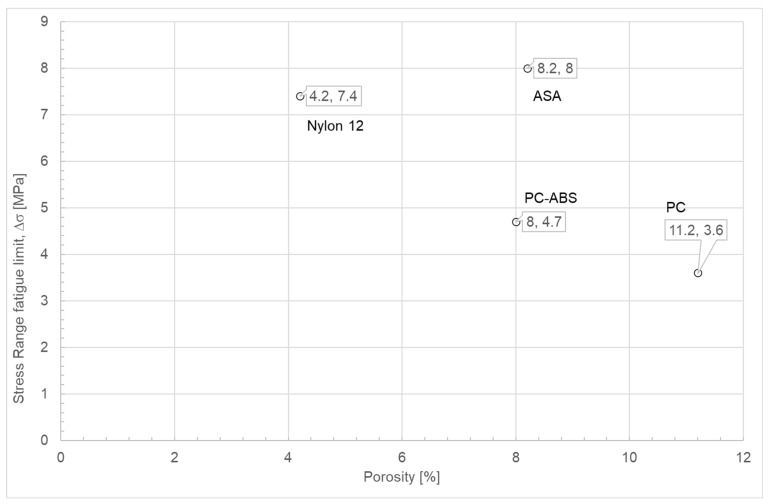
Fatigue limit versus level of porosity for the materials under study.

When comparing the porosity levels of each tested material (Table 9) with the stress–fatigue limit values (Table 11), it can be inferred that, in general, increasing porosity leads to lower fatigue limit stress values (Figure 13). At the same time, the porosity of the ASA and PC-ABS materials, which is comparable and around 8% for both materials, allows the inference of a higher fatigue resistance for ASA compared with PC-ABS, and with values similar to those of Nylon 12, even though Nylon 12 exhibited only 4.2% porosity. Thus, it appears that ASA would likely have the highest fatigue resistance if all materials had the same level of porosity.

Furthermore, PC-ABS has a significantly higher fatigue strength than PC, achieving infinite life at 15% × σR vs. 7% × σR for PC (see Table 11). This result may be counterintuitive, as PC typically shows higher strength than PC-ABS in *quasi*-static tests (Figure 2a). The micro-CT porosity data (PC: 11.2%, PC-ABS: 8.0%) partly explain this difference, once the higher porosity in PC (see Figure 10b), which seems to be highly sensitive to stress concentrations due to internal defects, increases the number and severity of internal stress concentrators. These internal defects are critical in fatigue, as voids act as initiation sites and accelerate crack propagation under cyclic loads, reducing the apparent fatigue limit. However, from the observation of Figure 10b,d, together with Figure 11c,d,g,h, it is also evident that, from the crack propagation mechanism point of view, PC tends to fail via more brittle microcrack linkage under cyclic loading, whereas PC-ABS may exhibit more tortuous and ductile crack propagation paths. Therefore, PC-ABS, as a polymer blend, appears to benefit from a more compliant ABS phase that dissipates cyclic energy more effectively than PC, blunts crack tips, and delays fatigue crack nucleation and propagation.

### 4.2. Engineering Implications and Biomedical Applications

For Nylon 12, its biocompatibility has already been confirmed in the literature. In the fatigue tests performed, specimens of this material withstood forces (fatigue limit) of up to 387.1 N. Therefore, according to [41], Nylon 12 could be used in dental applications, as it falls within the force ranges observed during mastication. Moreover, the experimentally determined fatigue limit stress of Nylon 12 was 7.4 MPa, which exceeds the typical range observed in cortical bone. In fact, in routine activities, bones generally experience stresses of approximately 4 MPa, a value that falls within the design range of Nylon 12. In parallel with the thermal analysis, it should be noted that the greatest degradation was observed in Nylon 12; therefore, its use at ambient or low temperatures is recommended. Furthermore, cytotoxicity, among other tests are required to confirm the suitability of Nylon 12 for bone-related applications.

Regarding ASA, research into its biocompatibility remains very limited. Although it exhibits infinite life at loads below 416 N and also falls within the values reported in [41], it is not possible to state that this polymer is a suitable option for dental applications. For the PC and PC-ABS polymers, compared with the other materials under study, they exhibit low fatigue resistance. As with ASA, the information available regarding their biocompatibility is still very limited.

### 4.3. Future Work

This study has advanced the understanding of the mechanical behaviour of polymeric parts produced by 3D printing. However, the application of such polymers in the biomechanical applications remains limited. Therefore, in vivo biocompatibility tests, cytotoxicity tests, long-term environmental degradation (including hydrolysis and enzymatic activity), wear resistance, stress shielding potential (particularly given the low modulus compared to cortical bone), osseointegration, and sterilisation compatibility tests are required to allow potential future biomechanical applications of the materials used in this investigation.

Future research should also determine the effects of fatigue test parameters, including frequency and stress ratio, on the mechanical behaviour of the studied materials. Additionally, conducting fatigue tests on 3D-printed models of internal and external prostheses, fabricated from the most promising materials identified in previous experiments, is recommended.

## Figures and Tables

**Table 2:** Technical specifications from Stratasys of the materials tested: ASA, PC, Nylon 12 and PC-ABS.

	ASA	PC	Nylon 12	PC-ABS
Commercial name	355-02141	355-02210	310-21800	310-20500
Grade	92.3 cu in.—Plus	92.3 cu in.—Plus	92.3 cu in.—Classic	92.3 cu in—Classic
Color	White	White	Black	Black
Glass transition temperature, Tg [°C]	104	142.5	34.03	105.3
Density [g/cm^3^]	1.08	1.20	1.01	1.10

**Table 3:** Printing parameters used for specimen manufacturing.

Printing orientation: XY
Layer thickness: 0.254 mm
Infill density: 100%
Raster angle: 45°/−45° Extrusion temperature: ASA (250 °C), PC (275 °C), Nylon 12 (255 °C), PC-ABS (265 °C) Build platform temperature: Nylon 12 (85 °C), ASA (100 °C), PC and PC-ABS (110 °C)

**Table 4:** Mechanical properties for each material tested.

Materials	Young’s Modulus [MPa]	Tensile Strength [MPa]	Ultimate Strain [%]
ASA	1423 ± 46.1	31.7 ± 0.3	11.2 ± 0.7
PC	1549 ± 132.7	57.1 ± 3.1	9.6 ± 0.8
Nylon 12	916 ± 16.9	37 ± 0.2	35.4 ± 2.1
PC-ABS	1230 ± 44.3	31.43 ± 0.7	5.6 ± 0.3

**Table 5:** Fatigue data for ASA.

Specimen (#)	Stress Range, Δσ [MPa]	Maximum Stress, $\sigma_{\max}$ [MPa]	Minimum Stress, $\sigma_{\min}$ [MPa]	Stress Amplitude, $\sigma_{a}$ [MPa]	Maximum Force, $F_{\max}$ [N]	Minimum Force, $F_{\min}$ [N]	Number of Cycles, $N_{f}$ [Cycles]
1	85% × σ_R_	28.63	1.43	13.6	1414.4	70.72	308
2	65% × σ_R_	21.89	1.09	10.4	1081.6	54.08	1519
3	30% × σ_R_	10.11	0.51	4.8	499.2	24.96	123,460
4	20% × σ_R_	6.74	0.34	3.2	332.8	16.64	1,000,000
5	25% × σ_R_	8.42	0.42	4	416	20.8	1,000,000
6	40% × σ_R_	13.47	0.67	6.4	665.6	33.28	29,658
7	27% × σ_R_	9.09	0.45	4.32	449.28	22.46	320,571
8	35% × σ_R_	11.79	0.59	5.6	582.4	29.12	47,978
9	27% × σ_R_	9.09	0.45	4.32	449.28	22.46	192,376
10	30% × σ_R_	10.11	0.51	4.8	499.2	24.96	90,640

**Table 6:** Fatigue data for PC.

Specimen (#)	Stress Range, Δσ [MPa]	Maximum Stress, $\sigma_{\max}$ [MPa]	Minimum Stress, $\sigma_{\min}$ [MPa]	Stress Amplitude, $\sigma_{a}$ [MPa]	Maximum Force, $F_{\max}$ [N]	Minimum Force, $F_{\min}$ [N]	Number of Cycles, $N_{f}$ [Cycles]
1	40% × σ_R_	21.35	1.07	10.14	1054.77	52.74	3368
2	25% × σ_R_	13.35	0.67	6.34	659.23	32.96	11,269
3	10% × σ_R_	5.34	0.27	2.54	263.69	13.19	182,224
4	5% × σ_R_	2.67	0.13	1.27	131.85	6.59	1,000,000
5	7% × σ_R_	3.74	0.19	1.78	184.58	9.23	1,000,000
6	9% × σ_R_	4.80	0.24	2.28	237.32	11.87	220,112
7	8% × σ_R_	4.27	0.21	2.03	210.95	10.55	834,070
8	8% × σ_R_	4.27	0.21	2.03	210.95	10.55	520,942
9	9% × σ_R_	4.80	0.24	2.28	237.32	11.87	299,132
10	8.5% × σ_R_	4.54	0.23	2.16	224.14	11.21	600,516

**Table 7:** Fatigue data for Nylon 12.

Specimen (#)	Stress Range, Δσ [MPa]	Maximum Stress, $\sigma_{\max}$ [MPa]	Minimum Stress, $\sigma_{\min}$ [MPa]	Stress Amplitude, $\sigma_{a}$ [MPa]	Maximum Force, $F_{\max}$ [N]	Minimum Force, $F_{\min}$ [N]	Number of Cycles, $N_{f}$ [Cycles]
1	85% × σ_R_	39.18	1.96	18.61	1935.52	96.78	1121
2	40% × σ_R_	18.44	0.92	8.76	910.83	45.54	29,657
3	20% × σ_R_	9.22	0.46	4.38	455.42	22.77	429,098
4	10% × σ_R_	4.61	0.23	2.19	227.71	11.39	1,000,000
5	15% × σ_R_	6.91	0.35	3.28	341.56	17.08	1,000,000
6	17% × σ_R_	7.84	0.39	3.72	387.10	19.36	1,000,000
7	19% × σ_R_	8.76	0.44	4.16	432.65	21.63	658,938
8	18% × σ_R_	8.29	0.42	3.94	409.87	20.49	951,076
9	19% × σ_R_	8.76	0.44	4.16	432.65	21.63	647,745
10	20% × σ_R_	9.22	0.46	4.38	455.42	22.77	395,066
11	30% × σ_R_	13.83	0.69	6.57	683.12	34.16	73,546
12	65% × σ_R_	29.96	1.49	14.23	1480.10	74.01	2551

**Table 8:** Fatigue data for PC-ABS.

Specimen (#)	Stress Range, Δσ [MPa]	Maximum Stress, $\sigma_{\max}$ [MPa]	Minimum Stress, $\sigma_{\min}$ [MPa]	Stress Amplitude, $\sigma_{a}$ [MPa]	Maximum Force, $F_{\max}$ [N]	Minimum Force, $F_{\min}$ [N]	Number of Cycles, $N_{f}$ [Cycles]
1	85% × σ_R_	28.18	1.41	13.39	1392.3	69.62	455
2	40% × σ_R_	13.26	0.66	6.3	655.2	32.76	13,927
3	15% × σ_R_	4.97	0.25	2.36	245.7	12.29	1,000,000
4	20% × σ_R_	6.63	0.33	3.15	327.6	16.38	256,682
5	17% × σ_R_	5.64	0.28	2.68	278.5	13.92	688,867
6	16% × σ_R_	5.31	0.27	2.52	262.1	13.10	964,381
7	17% × σ_R_	5.64	0.28	2.68	278.46	13.92	583,107
8	18% × σ_R_	5.97	0.30	2.84	294.84	14.74	441,289
9	25% × σ_R_	8.29	0.42	3.94	409.5	20.48	83,566
10	30% × σ_R_	9.95	0.50	4.73	491.4	24.57	35,769

**Table 9:** Porosity measured from the micro-CT analyses.

Material	Volume of Solid Material (μm^3^)	Percentage of the Identified Volume	Porosity
ASA	$5.6512\times 10^{10}$	91.801%	8.199%
PC	$5.4656\times 10^{10}$	88.786%	11.214%
Nylon 12	$5.8951\times 10^{10}$	95.763%	4.237%
PC-ABS	$5.6660\times 10^{10}$	92.041%	7.959%

**Table 10:** Results from the TGA and DSC analysis. Temperatures at 5, 10 and 50% mass loss, T_5_%, T_10_%, and T_50_%, respectively. Onset decomposition temperature, T_onset_. Glass transition temperature, Tg. Melting temperature, Tm.

Material	Initial Mass (mg)	Degradation (%)	Final Mass (mg)	Mass Loss Rate (%/min)	Temperature of Degradation (°C)	T_5_% (°C)	T_10_% (°C)	T_50_% (°C)	T_onset_ (°C)	Tg (°C)	Tm (°C)
ASA	5.992	89.63	0.621	−20.631	415.24	360.73	380.59	413.37	370	100	-
PC	6.324	78.83	1.339	−14.119	509.47	462.82	473.45	509.51	460	150	-
Nylon 12	7.833	98.49	0.118	−24.166	460.23	417.37	426.33	454.11	420	45	178
PC-ABS	8.285	92.03	0.661	−18.802	451.02	396.59	410.93	445.19	400	105; 145	-

**Table 11:** Fatigue limits, Δσ.

Material	Stress Range	Stress Range, Δσ [MPa]	Maximum Force, F_max_ [N]
ASA	$20\%\times \sigma_{R}$	6.4	332.8
	$25\%\times \sigma_{R}$	8	416
PC	$5\%\times \sigma_{R}$	2.5	131.9
	$7\%\times \sigma_{R}$	3.6	184.6
Nylon 12	$10\%\times \sigma_{R}$	4.4	227.7
	$15\%\times \sigma_{R}$	6.6	341.6
	$17\%\times \sigma_{R}$	7.4	387.1
PC-ABS	$15\%\times \sigma_{R}$	4.7	245.7

## Data Availability

The raw data supporting the conclusions of this article will be made available by the authors on request.
